# The role of spatial mobility in malaria transmission in the Brazilian Amazon: The case of Porto Velho municipality, Rondônia, Brazil (2010-2012)

**DOI:** 10.1371/journal.pone.0172330

**Published:** 2017-02-21

**Authors:** Jussara Rafael Angelo, Tony Hiroshi Katsuragawa, Paulo Chagastelles Sabroza, Lino Augusto Sander de Carvalho, Luiz Hildebrando Pereira da Silva, Carlos Afonso Nobre

**Affiliations:** 1 Departamento de Endemias Samuel Pessoa, Escola Nacional de Saúde Pública, Fundação Oswaldo Cruz (ENSP/FIOCRUZ), Rio de Janeiro, Rio de Janeiro, Brasil; 2 Fundação Oswaldo Cruz - Rondônia (Fiocruz-RO), Porto Velho, Rondônia, Brasil; 3 Instituto Nacional de Pesquisas Espaciais (INPE), São José dos Campos, São Paulo, Brasil; Universidade de Brasilia, BRAZIL

## Abstract

**Background:**

This study aims to describe the role of mobility in malaria transmission by discussing recent changes in population movements in the Brazilian Amazon and developing a flow map of disease transmission in this region.

**Methodology/Principal findings:**

This study presents a descriptive analysis using an ecological approach on regional and local scales. The study location was the municipality of Porto Velho, which is the capital of Rondônia state, Brazil. Our dataset was obtained from the official health database, the population census and an environmental database. During 2000–2007 and 2007–2010, the Porto Velho municipality had an annual population growth of 1.42% and 5.07%, respectively. This population growth can be attributed to migration, which was driven by the construction of the Madeira River hydroelectric complex. From 2010 to 2012, 63,899 malaria-positive slides were reported for residents of Porto Velho municipality; 92% of the identified samples were autochthonous, and 8% were allochthonous. The flow map of patients' movements between residential areas and areas of suspected infection showed two patterns of malaria transmission: 1) commuting between residential areas and the Jirau hydropower dam reservoir, and 2) movements between urban areas and farms and resorts in rural areas. It was also observed that areas with greater occurrences of malaria were characterized by a low rate of deforestation.

**Conclusions:**

The Porto Velho municipality exhibits high malaria endemicity and plays an important role in disseminating the parasite to other municipalities in the Amazon and even to non-endemic areas of the country. Migration remains an important factor for the occurrence of malaria. However, due to recent changes in human occupation of the Brazilian Amazon, characterized by intense expansion of transportation networks, commuting has also become an important factor in malaria transmission. The magnitude of this change necessitates a new model to explain malaria transmission in the Brazilian Amazon.

## Introduction

Malaria disease events in the Brazilian Amazon present a spatial pattern defined by environmental and socio-spatial factors, including vector density, land cover dynamics, population dynamics and economic activities such as mining and the construction of hydroelectric dams, and the capacity of health services to control the disease [1] [2]. One of the socio-spatial processes that allow population groups to become more vulnerable to diseases is population mobility (PM). PM plays an important role in disease transmission by increasing the number of susceptible people, enabling the transfer of previously unexposed population groups to an endemic area, which results in a greater occurrence of severe disease or death [3].

Although PM data are scarce and hence few empirical studies of PM have been published, several authors have highlighted the importance of PM in the occurrence of health problems, including, specifically, malaria [1] [2] [4] [5] [6] [7]. PM not only results in human contact with novel pathogens but also promotes the spread of other pathogens to new areas. Furthermore, in areas where a disease has been successfully controlled, PM contributes to disease re-emergence and hampers efforts to maintain disease control [6] [7]. PM is responsible for increasing the frequency of social contacts that also can increase the basic reproductive rate of a pathogen (R_0_). R_0_ is defined as the average number of secondary infections produced per infected individual over the course of its infectious period, when introduced into a totally susceptible population [8]. Moreover, PM contributes to the circulation and introduction or reintroduction of new variants (strains) of parasites in endemic areas, and such new exposures or increased levels of exposure may cause outbreaks and increase the frequency of more severe cases.

Until the late 1980s, PM in the Brazilian Amazon was largely driven by migration flows from different regions of the country, especially from the Southeast and the Northeast. These migrants were attracted by the expansion of the agricultural frontier and also by development projects such as rural settlements, mining and hydropower construction [9]. The classic and pioneering studies by Marques & Pinheiro [10] and Marques [11] linked the spread of malaria to mobility in Brazil. The results showed that in 1980, displacement patterns of malaria cases (1) within the Amazon and (2) from the Amazon to the rest of the country were very important epidemiologically. Mobility within the Amazon was due to migration, which was driven by the pursuit of land in rural settlements, minerals in mining developments or employment opportunities provided by hydropower projects. The second mobility pattern resulted from the return of these migrants to their places of origin [10] [11].

Over the last 20 years, the Brazilian Amazon has gone through many transformations that have caused new PM patterns to emerge. One of the most relevant transformations has been the increase in connectivity between places; this increased connectivity has enabled the region to communicate far more both within the country and with locations abroad. In addition, the substantial growth rate of urban populations in the Amazon (one of the highest urban growth rates in the country) has emerged big cities such as Manaus and Belém but, mainly several medium-sized cities which have played important roles in new configuration of Amazonian territory [12] [13] [14]. Furthermore, the emergence of new economic activities in urban centers and the growth of previously occupied areas, combined with increasing urbanization and the development of a road network, have contributed to the increase in net economic flows and, consequently, the increase in PM in its different forms [14]. In light of this new context in the Amazon, this work attempts to describe the role of population mobility on malaria transmission. The analysis is limited to 2010–2012 patterns, however, the discussion compares our results with historical patterns of malaria transmission in the region, indicating that new types of mobility linked to developmental process which are shifting malaria transmission in the Brazilian Amazon. While other studies have found that mobility plays a role in the transmission and increasing occurrence of diseases such as malaria, there is little work done to consider the specific spatial and temporal modalities of population mobility. Thus, we seek to examine the relationships between migration, commuting and general mobility within the municipality of Porto Velho in Rondônia State.

## Material and methods

### Study area

The study area was the Porto Velho municipality, which is the capital and largest urban center in the state of Rondônia. This region has been characterized by important changes in its spatial organization in recent years. Historically, large-scale infrastructure projects have been pursued in this municipality. In 2008, two very large infrastructure projects were initiated in the area, namely the Jirau and Santo Antônio hydroelectric dams on the Madeira River. These projects have boosted the region’s economy and changes in the demographic dynamics of the municipality by attracting an estimated 25 thousand temporary laborers to the region [15] (S1 Fig).

### Study design

This ecological/aggregate and descriptive study was conducted to understand the changing patterns of PM and their relationship with the occurrence of malaria. To identify the flows of malaria transmission in the municipality of Porto Velho on local and regional scales, data were obtained from the Malaria National Information System (Sistema de Informação da Malária—SIVEP—Malaria), the National Information System for Compulsory Notification (Sistema de Informação de Doenças de Notificação Compulsória—SINAN), and the Ministry of Health. Data from 2010 to 2012 were analyzed. It is important to highlight that all patient records/ and information were anonymized and de-identified prior to the analysis. The demographic data examined in this study were from the 2000 and 2010 Census, the population count of 2007, and the National Domicile Sampling Research (Pesquisa Nacional de Amostra por Domicilio—PNAD) for 2010. All demographic datasets belong to the Brasilian Geography and Statistics Institute (Instituto Brasileiro de Geografia e Estatistica—IBGE) [16]. This data was used to observe and quantify demographic trends, including population growth and migration.

Malaria cases were classified as autochthonous, allochthonous or migrant. Autochthonous cases were those in which the place of infection was the municipality of residence (Porto Velho municipality); allochthonous, or imported, cases were those of individual residing in Porto Velho who were the infected in another municipality; and migrant cases refer to cases in which individuals were infected in Porto Velho but lived in another municipality [17]. It is necessary to make clear that SIVEP-Malaria is a system that store notifications from endemical areas and SINAN is responsible to notify malaria cases in non-endemical areas. So the migrants cases can be from endemical areas or not non-endemical areas.

Flow maps between the place of residence of the malaria patient (origin) and the likely site of infection (destination) were generated for residents in the municipality of Porto Velho. The unit of analysis used was the village location registered in the SIVEP—Malaria system, whose territorial base was organized in partnership with the Instituto de Pesquisa em Patologias Tropicais (Ipepatro/Fiocruz—Rondônia) and field supervisors of the Center for Zoonosis Control from the Porto Velho municipality.

The flow map was classified by the natural breaks classification method. It is important to highlight that the site of infection is the place declared by the patient where the malaria transmission probability occurred; it is information related a perception of risk of patient.

In this work, methods and technics of spatial analysis from Health Geography were used which provided great contributions to understanding of spatial and temporal distribution of health problems and social and environmental determination of the health-disease process, in particular Graph Theory and Geographic Information System (GIS).

The flows are displayed as graphs, which are mathematical representations of the data and consist of a set of points and a set of lines that connect the points in pairs. The points are called vertices, and the connections are called edges [18].

In our work, the vertices represent the rural areas (localities) of Porto Velho municipality, and the edges are the flow of people infected with malaria during their displacements between these locations. To present the data more clearly, we chose to use weighted graphs. Weighted graphs associate a numerical value with each edge. In this case, the numerical value refers to the number of people who became infected with malaria (S2 Fig).

To facilitate visualizing the data more clearly, only patients whose place of residence was different from the village in which they were most likely infected and flows whose frequency was greater than or equal to 5 occurrences were mapped. Furthermore, the data of the localities belonging to the Porto Velho urban area were grouped into a single centroid.

The open source platform Terraview (version 4.2.0), developed by the National Institute for Space Research, was used to build the presented maps. TerraView geographical data and operational functions are based in a collaborative open source GIS library called TerraLib. In addition to visualization tools, TerraView provides a set of analysis tools that include database queries, data analysis and a series of plug-ins, and particularly the *flux plug* in which was used for fluxes maps [19].

Malaria flow patterns can be represented by a network and described by Graph Theory metrics. The statistical analysis of the malaria transmission graphs network was performed using the Social Network Visualizer (SocNet-V 1.5), additionally, an open-source software developed by Kalamaras was used [20]. Four metrics were calculated for each network node network: a degree (in-degree and out-degree), a centrality degree, a prestige degree and a clustering coefficient. The first three metrics are centrality metrics, it is an attempt to quantify how central each node is inside the network and examine the ties attached to that node and its geodesic distances to other nodes. The clustering coefficient of a node quantifies how close the node and its neighbors are to a click. A click is a group of actors that interacts with each other more regularly and intensely than others on the same network [21]

Data from land cover and land changes were also used to discuss the results, since previous studies have highlighted the importance of land-use in malaria occurrence in the Amazon [2] [22]. Land cover data and changes in land use were obtained from the Terraclass project organized by INPE [23]. The following land-use categories were included in maps that it was used in this study: “pasture”, “forest”, “urban area”, “mining’, “second growth”, “water”, ‘occupation mosaic’, “no observed” and “others”.

It is important to highlight the meaning of the three last categories: The category “occupation mosaic” refers to areas represented by different types of land cover that, due to the spatial resolution of satellite images, could not be distinguished from the components. In this class, family farming is mixed with the pasture of traditional livestock. On the other hand the category “others” includes areas that have a pattern of differentiated coverage of all project classes, such as rock outcrops, river beaches and sandbars. "No observed" involves areas that the interpretation was assumed impossible due to the presence of clouds in the image [23]. Data on deforestation detected between 2010 and 2012 from the PRODES database were also included [24].

Data from the National Institute of Colonization and Agrarian Reform was used to map the new rural settlements which were installed in Porto Velho municipality. The category “New settlement” was considered the rural settlements whose occupation began 10 years before the period analyzed (2010–2012) [4]. “Special interest area” was assumed as indigenous and protected areas. The “special interest areas” were assumed as indigenous and protected areas.

### Ethical considerations

This study was approved by the Research Ethics Committee of the Instituto em Ciência e Tecnologia Campus São José dos Campos—UNESP, CAAE (approval no. 23222413.2.0000.0077).

## Results

In 2000, the municipality of Porto Velho had a population of 334,661. By 2007, the population had increased to 369,345, and by 2010, to 428,527 inhabitants. This corresponds to a geometric growth rate of 1.42% per year in the first period (2000–2007) and 5.07% in the second period (2007–2010). These growth rates were higher than the Rondônia state population growth rate averages of 0.75% and 2.43% for the same periods, respectively.

Survey data taken from PNAD shows that 82,810 people moved to Porto Velho in the ten year period from 2000 to 2010 and that approximately 90% of that population growth resulted from migration (S1 Table), primarily from the states of Maranhão (MA, 6.58%), Pará (PA, 6.65%), São Paulo (SP, 6.80%), Mato Grosso (MT, 7.59%), Acre (AC, 10,23%) and Amazonas (AM, 13.60%).

From 2010 to 2012, 63,899 malaria-positive slides were reported among residents of the Porto Velho municipality in Rondônia state territory. Among those cases, 92% were autochthonous, whereas 8% were allochthonous.

Of allochthonous cases, 81% were concentrated in municipalities that share a border with Porto Velho. The municipality of Candeias do Jamari, at a distance of 20 km from the Porto Velho urban area, and the municipality of Canutama, on the banks of the Purus River in the Amazonas state, were responsible for the majority of allochthonous cases, with 42% and 24%, respectively (S1 and S3 Figs). S3 Fig presents municipalities that reported more than 20 allochthonous cases. The remaining allochthonous cases were distributed among 104 municipalities in the Rondônia, Acre and Amazonas states.

For total of 2166 migrant cases from endemic areas being observed that the municipalities of origin were concentrated primarily in the Amazonas state. The municipalities of Canutama (AM), Manaus (AM), Barcelos (AM) and Humaitá (AM) accounted for approximately 78% of reported migrant cases during the 2010–2012 period (S1 and S4 Figs). As in S3 Fig, only the municipalities with more than 20 migrant cases are shown in S4 Fig. The remaining cases, which represent 6% of all migrant cases, were distributed among 187 municipalities. During the 2010–2012 period, the municipality of Porto Velho had the highest percentage of migrant cases from non-endemic areas in Brazil. Of the migrant cases, 17.45% (550 cases) were infected in the Porto Velho municipality; this percentage is three times the percentage (4.98%) infected in the municipality with the second highest frequency, Manaus, which is the capital of the Amazonas state. It is important to note that 32% of the migrant cases living outside endemic areas are from abroad. The analysis of population flows between places of residence and probable places of infection within the Porto Velho municipality showed that the flows where the most infections occurred were the following: 1) Acampamento Caldeirão to Usina de Jirau; 2) Jaci-Paraná to Usina de Jirau; 3) urban area of Porto Velho to Bacia Leiteira; 4) urban area of Porto Velho to Belmonte; and 5) urban area of Porto Velho to Balneário Areia Branca (S5 and S6 Figs).

Based on the spatial configurations of the network (S5 and S6 Figs), the location of dam operations, and knowledge of local context, transmission appears to follow two patterns. The first pattern is caused by commuting related to work displacement; in this pattern, employees of Jirau hydropower dam that live in urban areas or work-camps become infected during working hours. The second pattern is driven by transmission that occurs in farms and rural areas near the urban area of Porto Velho municipality.

The results from the metrics used to identify the properties of malaria transmission network also confirm these explanations.

The metric *in degree* showed that the localities Usina de Jirau, Acampamento Caldeirão and Jacy Paraná were the localities that received the greatest quantity of edges from other localities, namely 143, 76 and 64 edges, respectively. The same pattern was observed for the metric *prestige degree*, which showed that these localities presented cases of malaria (Usina de Jirau—11.4%, Acampamento Caldeirão—5.93% and Jacy Paraná 5.00%).

On the other hand, the results obtained for the metric *out degree* metric shows that the urban area of Porto Velho sent the highest number of edges to other parts of the municipality (950 edges). The urban area also registered the highest value for the metric *centrality degree*, which showed that 74.2% of the malaria cases are from urban area.

It must be mentioned that the metrics *in-degree* and *out-degree* did not take into account the weight of the network and only considered the number of edges that the node received (in degree) and the node sent (Out degree). A different behavior is stated by the *centrality degree* and *prestige degree* metrics, which considers the weights of network.

The aim of using the *clustering coefficient* metric was to identify the transitivity phenomenon, through the clustering of triangles in the network (set of three vertices connected to each other). The results showed that the Acampamento Caldeirão locality presented the highest transitivity in the malaria transmission network, which shows that this locality and another vertex that shares a neighbor node with this locality has a high probability of being linked to each other (S2 Table).

In the 2010–2012 period, in the municipality of Porto Velho, the areas more deforested were south and southeast. However, in these regions, the malaria was primarily detected in the oldest areas of occupation and the areas of special interest (indigenous areas and protected areas). Besides, areas surrounding the urban area of Porto Velho and the Jirau Hydropower reservoir area, places with high disease flow, had small areas deforested (S6 and S7 Figs).

## Discussion

The significant increase in the population growth rate in the 2007–2010 period in the municipality of Porto Velho (5.07% per year) can be attributed primarily to the construction and subsequent operation stage at the Santo Antônio and Jirau hydroelectric dams on the banks of the Madeira River. Construction of these dams began in 2008, and they began operating in 2010.

The municipality of Porto Velho plays two important roles in malaria transmission. First, the municipality is highly receptive to malaria, and malaria is highly endemic, as evidenced by the high percentage of cases that were reported as autochthonous (92%). Second, the municipality disperses the disease to other municipalities in the Amazon and even to non-endemic areas of the country. On the other hand, employment or other activities, such as tourism, increase the movements of Porto Velho residents to neighboring municipalities, enabling the occurrence of imported cases of malaria (8% of cases are allochthonous). In spite of their lower frequency, these cases are of critical epidemiological importance because they may be responsible for the introduction of new variants of the parasite in the municipality of Porto Velho.

Currently, population migration to municipalities in the Amazon Region still occurs because of large economic development projects that provide employment opportunities. Despite the reduction in migration flows that took place in the 1980s [25], migration is still a substantial factor driving malaria occurrence in the municipality of Porto Velho, as it increases the number of susceptible individuals. The present results show that a significant proportion of the population growth from 2007 to 2010 was related to migration and was driven by the construction and subsequent operation of hydropower dams on the Madeira River. There is, however, a difference between the migration observed in the 1980s and the population movements observed today. In the past, migration was related to government projects for agricultural settlements, and migrants settled permanently at their destinations. In contrast, the current migration pattern in this region consists of temporary movements motivated by temporary work [26].

According to Alves and Junior [27], different migration flows can be observed during the three phases of implementation of a hydropower dam. During the planning phase, skilled labor is initially mobilized by the hiring of multidisciplinary technical teams for engineering and environmental studies. Next is the construction phase of the project; this phase is considered to have the greatest impact on the region because of the large mobilization of manpower required for construction for both the project itself and the infrastructure needed for the project. The end of this phase is characterized by a collapse in the demand for temporary labor and produces large-scale unemployment. Finally, only a skeleton crew remains to fill the reservoir and maintain normal operation of the hydropower dam.

A network of connections within the Porto Velho municipality integrates remote areas that are difficult to access with urban areas and enhances disease transmission in all regions (S5 Fig). This network resulted from an intensification of subregional networks that connect rural areas to urban areas. This intensification has been caused by improvements in transportation and communication systems and by rural populations seeking employment opportunities and services offered in the city [28].

The flow maps between place of residence and location of infection reveal a spatial pattern of disease transmission that results from two types of commuting: first, by workers who were infected in hydropower dam regions; second, by residents of urban areas to rural areas surrounding the Porto Velho urban area (S6 Fig and S2 Table).

It is important to highlight that the migration process occurs when population groups switch places with residence in a definitive temporal perspective while the commuting refers the movement that occurs, “daily,” between the place of residence and the place of work. On the other hand the, “mobility,” is a general term used to for any population movement in the territory [29].

The infection of a large population flow at the Jirau hydropower dam can be attributed to the prevailing receptivity in the area and also to the presence of specific population groups exposed to the disease. *Anopheles* mosquitos are present in these areas at high densities because of environmental changes resulting from the large-scale economic projects. It is also important to note the high rates of exposure of large numbers of workers who commute daily to this region [30].

The primary malaria vector in Brazil is *Anopheles darlingi*, which lives at low altitudes and chooses large, lentic water bodies with little or no current, low salinity and near neutral pH [31] [32]. Larvae are usually encountered on water body margins—primarily in deep, clean, slightly turbid and sunny or partially shaded water, hiding in vegetation or debris [32] [33]. Human alterations of the land can synergize with natural characteristics to increase the numbers of suitable breeding sites and, consequently, vector density. When comparing vector densities across areas subjected to human interference, such as roads and manmade flooded areas from dam construction, higher vector densities were observed in clear water and also within forest areas [31] [34]; these findings show that land use and land cover changes play a major role in developing vector receptivity conditions.

The flow patterns between urban areas and rural areas surrounding the Porto Velho urban area are related to tourism. An example of this is the flow directed to Balneário Areia Branca, a lake that people visit on the weekends to go swimming. The possibility that some people are "multilocalized," i.e., that they keep two residences, should also be considered.

Pinedo-Vasquez et al. [35] analyzed the migrant population that had moved from rural areas to urban areas in Macapá, the capital of Amapá state, and found that many poor families or slum dwellers are "multilocalized"; they maintain a home and conduct economic activities not only in a rural community but also in one of the suburbs or slums surrounding the city. According to the authors of this study, the majority of these families have at least one member who stays in the urban area for extended periods of time, while other members move between the rural community and the city.

An analysis of the land use and cover map (S7 Fig) and of the flow map of malaria transmission (S5 and S6 Figs) suggests a new pattern for malaria occurrence in the Brazilian Amazon. This new pattern can be explained by a model that is very different from the frontier malaria model, which is currently used in most malaria studies conducted in the Brazilian Amazon. The frontier malaria pattern is directly related to the development of agricultural settlements in the Brazilian Amazon. The first years of occupation are characterized by intense deforestation and therefore an increase in mosquito breeding; deforestation produces pits that accumulate water and become suitable for *Anopheles* proliferation. Furthermore, poor housing, which is usually improvised with cardboard, plastic or even palm leaves, causes the population to become increasingly exposed and makes spraying at home unfeasible. Because of these characteristics of the early stages of the occupation process, a malaria outbreak occurs; the incidence rate of this outbreak eventually declines and stabilizes at an endemic level 8–10 years later. This behavior is largely explained by several factors, including urbanization, the establishment of pastures and crops that limit receptive conditions for the vector, and improvements in socioeconomic conditions. Moreover, in the early years of occupation, no established healthcare system is available to reduce transmission rates or offer treatment to patients [2] [36] [37] [38].

The frontier malaria model may no longer be able to fully explain the occurrence of malaria in the municipality of Porto Velho. Although the municipality has a large migratory flux, the areas with a high frequency of malaria cases possess neither high deforestation rates nor recent agricultural settlements (S5–S7 Figs).

Because of the new historical context of human occupation of the Brazilian Amazon, characterized by marked connectivity and greater mobility, it would be important to improve our understanding of malaria transmission in Porto Velho. Migration remains an important factor that drives malaria transmission, as large development projects attract workers and people seeking to improve their lives. However, a portion of this migratory flow is temporary and is primarily composed of workers that stay in the municipality only during construction of the hydropower dam. Commuting, for work, tourism, or travel between two residences, then emerges as an important factor linking land use and the endemic circulation of malaria.

It would also be important to incorporate an understanding of the role of mobility in malaria transmission into the health system’s approach to malaria. Current strategies for malaria control reflect a different historical context and, therefore, should be revised in light of more recent changes in the social and environmental determinants of disease.

Because population migration patterns in the Amazon have changed, it is necessary to conduct studies at local scales to identify and characterize new patterns of mobility and their influence on malaria transmission. Besides, to strengthen the malaria epidemiological surveillance in Rondônia state is mandatory to improve the communication among state health departments responsible for malaria control.

The findings of this study emphasize the complexity of factors involved in the dynamics of malaria transmission in the Brazilian Amazon. This study identifies spatial mobility, especially in the form of commuting, as an important determinant of malaria transmission in Porto Velho, because it is the capital and most important economic center of the state. This transmission pattern occurs, particularly, in municipalities that present consolidated urban areas and that act as a node of urban network, that is, are central areas to trade and urban services, and therefore attract people from surrounding.

Even though the findings of this study had contributed to understanding the role of mobility in malaria transmission in Brazil, we recognize the limitations of this work, mostly due to the lack of available data. Although both SINAN and SIVEP are important sources of information about malaria, they are secondary data sources which have well-known limitations regarding completeness, in particular for discriminating place of residence and infection. We point out, the necessity to construct databases that provide information about daily movements of population in the Brazilian territory.

In conclusion, we should note that, in a holistic approach to understanding the social and environmental determinants of malaria, it is necessary to integrate knowledge from diverse scientific fields. Such a combined approach will not only provide support to local health services treating this disease but also produce better working and living conditions, which will, in turn, result in better health.

## Supporting information

S1 Fig Location of the local administrative area of Porto Velho municipality, Rondônia State, Brazil. (TIF)

S2 Fig Representation of a weighted graph. (TIFF)

S3 Fig Allochthonous cases of malaria in Porto Velho, by municipality of residence, 2010–2012. (TIF)

S4 Fig Migrant cases of malaria contracted in Porto Velho and reported in endemic areas, by municipality of residence, 2010–2012. (TIF)

S5 Fig Flow map showing the connections between villages of residence and probable villages of infection. (TIFF)

S6 Fig Map of major flows between villages of residence and probable villages of infection in the Porto Velho municipality. (TIFF)

S7 Fig Land use and land cover in the Porto Velho municipality. (TIFF)

S1 Table Population that has inhabited the Porto Velho municipality for an uninterrupted time period of less than 10 years, arranged by the duration of residence. (DOCX)

S2 Table Statistical properties of major flows of transmission of malaria in Porto Velho municipality. (DOCX)
