# Supplementary figures and images for: The role of spatial mobility in malaria transmission in the Brazilian Amazon: The case of Porto Velho municipality, Rondônia, Brazil (2010-2012)
Source: PLoS One. 2017 Feb 21;12(2):e0172330. doi: 10.1371/journal.pone.0172330 (PMC5319790; doi:10.1371/journal.pone.0172330)

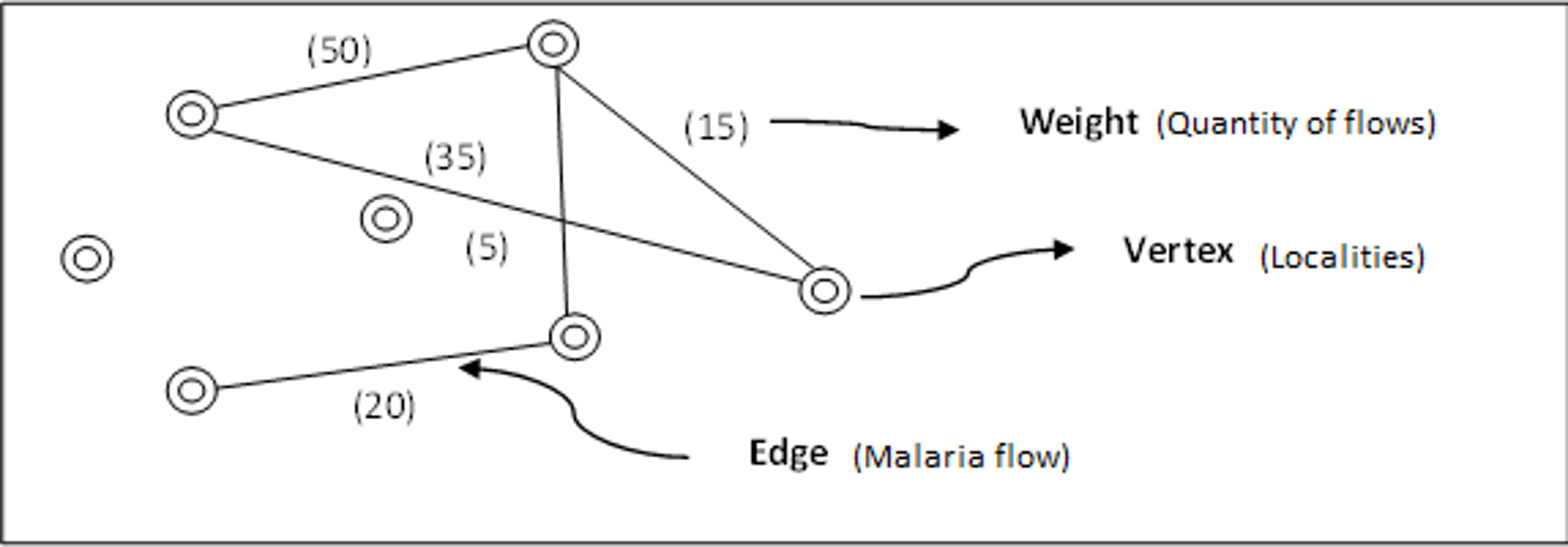

Supplement: S2 Fig — (TIFF) [file pone.0172330.s002.tiff]

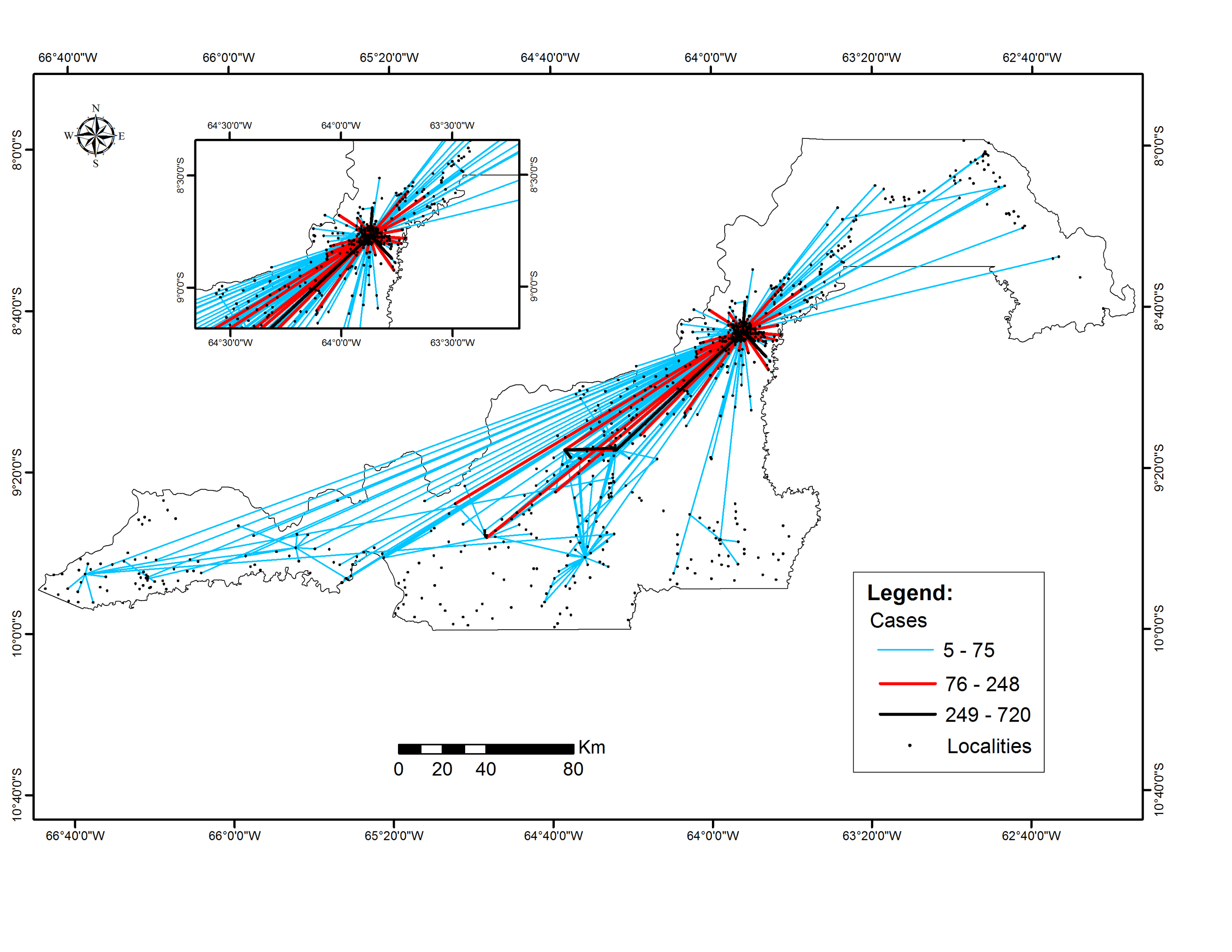

Supplement: S5 Fig — (TIFF) [file pone.0172330.s005.tiff]

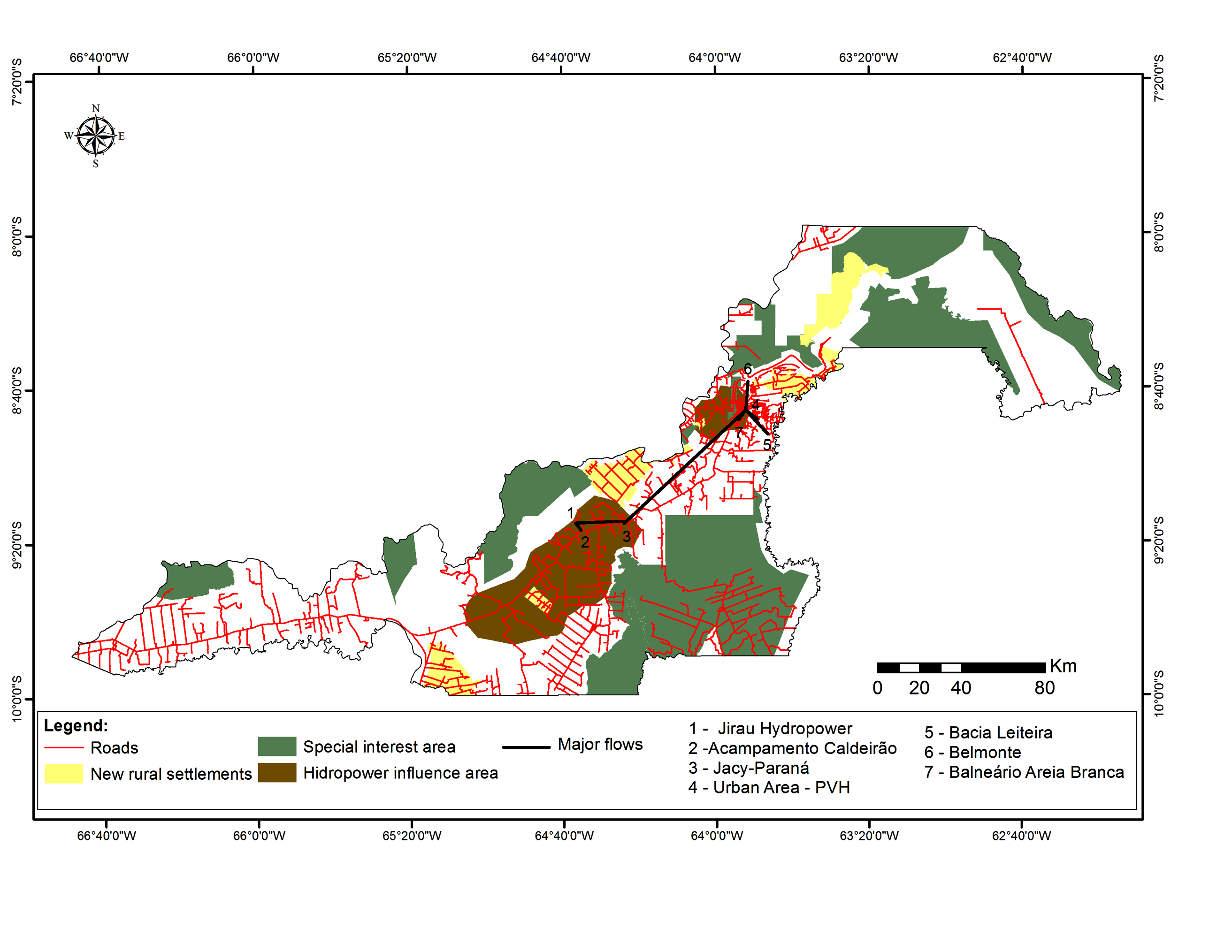

Supplement: S6 Fig — (TIFF) [file pone.0172330.s006.tiff]

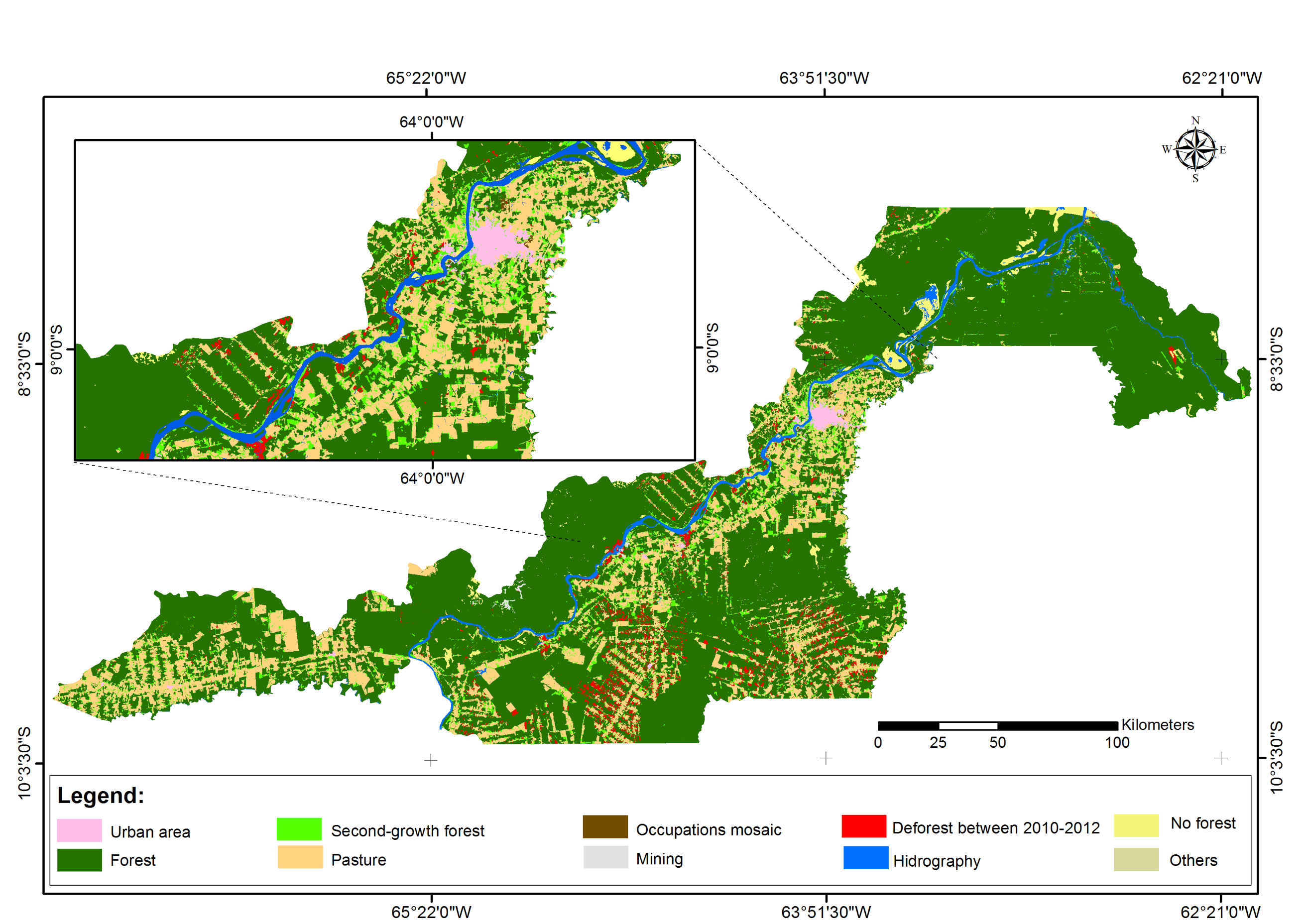

Supplement: S7 Fig — (TIFF) [file pone.0172330.s007.tiff]
