# Supplementary material for: The role of spatial mobility in malaria transmission in the Brazilian Amazon: The case of Porto Velho municipality, Rondônia, Brazil (2010-2012)
Source: PLoS One. 2017 Feb 21;12(2):e0172330. doi: 10.1371/journal.pone.0172330 (PMC5319790; doi:10.1371/journal.pone.0172330)
Supplement: S1 Table — (DOCX) [file pone.0172330.s008.docx]

Table S1 – Population that has inhabited the Porto Velho municipality for an uninterrupted time period of less than 10 years, arranged by the duration of residence.

| Duration (year) | Number of People | Percentage (%) |
| --- | --- | --- |
| < 1 | 19, 582 | 23.65 |
| 1 - 2 | 26, 589 | 32.11 |
| 3 - 5 | 18, 380 | 22.20 |
| 6 - 9 | 18, 258 | 22.05 |
| Total | 82, 810 | 100.00 |
