# Supplementary material for: The role of spatial mobility in malaria transmission in the Brazilian Amazon: The case of Porto Velho municipality, Rondônia, Brazil (2010-2012)
Source: PLoS One. 2017 Feb 21;12(2):e0172330. doi: 10.1371/journal.pone.0172330 (PMC5319790; doi:10.1371/journal.pone.0172330)
Supplement: S2 Table — (DOCX) [file pone.0172330.s009.docx]

Table S2 – Statistical properties of major flows of transmission of malaria in Porto Velho municipality.

|  | In degree | Out degree | Centrality  degree (%) | Prestige  degree (%) | Clustering  coefficient* |
| --- | --- | --- | --- | --- | --- |
| Acampamento Caldeirão | 76 | 0 | 0.0 | 5.93 | 0.75 |
| Usina de Jirau | 146 | 33 | 2.58 | 11.4 | 0.21264 |
| Jacy Parana | 64 | 19 | 1.48 | 5.0 | 0.17619 |
| Area Urbana | 3 | 950 | 74.2 | 0.234 | 0.00267 |
| Bacia Leiteira | 37 | 0 | 0.0 | 2.89 | 0.0 |
| Belmonte | 33 | 1 | 0.0781 | 2.58 | 0.0 |
| Balneario Areia Branca | 5 | 0 | 0.0 | 2.34 | 0.0 |
